# "The Most Gentle of Lethal Methods": The Question of Retained Consciousness Following Decapitation

**DOI:** 10.7759/cureus.33830

**Published:** 2023-01-16

**Authors:** Matthew D Turner

**Affiliations:** 1 Hospital-Based Medicine, Madigan Army Medical Center, Lakewood, USA

**Keywords:** brain trauma injury, decapitation, alterations in consciousness, french revolution, guillotine, history of medical sciences

## Abstract

Since the development of the infamous guillotine in the French Revolution, physicians have debated how long consciousness persists in decapitated heads. Fueled by anecdotes of severed heads that blink, blush, and appear to retain intelligence, numerous experiments have investigated this macabre subject for nearly 250 years. In this paper, we examine the evidence, both historical and modern, and ultimately conclude that, while the truth may never be fully known, all evidence appears to indicate that loss of consciousness appears to occur within seconds of decapitation. The rumors that circulated through the European consciousness during the Terror of the French Revolution appear to be just that - curious urban legends from an awed and terrified public.

## Introduction and background

For over 200 years, the guillotine of the French Revolution has been a subject of fascination and terror. Made infamous by novels such as *A Tale of Two Cities, *the guillotine today is a symbol of oppression and death. As thousands perished beneath its blade, rumors soon spread that those unlucky enough to be beheaded by the device did not experience the painless, instantaneous death that the revolutionary government promised - and that the condemned continued to display signs of life and awareness even after they had been decapitated. This fear became a source of great controversy for the physicians of the day, and the debate has even continued to the modern age - though the debate is now centered on animal models for medical experimentation rather than humans.

## Review

In his 1957 essay on capital punishment,* Réflexions sur la guillotine*, the French philosopher Albert Camus described the guillotine thusly: “…under a night sky, one of the executioners will finally seize you by the seat of your pants and throw you horizontally on a board while another will steady your head in the lunette and a third will let fall from a height of seven feet a hundred-and-twenty-pound blade that will slice off your head like a razor” [1]. It was a grim fate for the condemned, and for decades, rumors had persisted that it was an even crueler death than it seemed. Two generations earlier, on the 28th of June, 1905, Dr. Jacques Beaurieux witnessed the execution of a condemned criminal by guillotine. Curious at the head’s twitching eyes and spasming lips, the doctor performed a morbid experiment. “Languille!” he called out the criminal’s name. To his astonishment, the eyes lifted and “…fixed in a precise fashion on mind and the pupils adjusted… I had the impression that living eyes were looking at me” [2]. Dr. Beaurieux’s experiment was merely one in a long line of fierce arguments and counter-arguments that raged within the European medical community for generations: *Do decapitated heads retain consciousness?*

“The blade hisses, the head falls, blood spurts, the man exists no more,” Dr. Joseph-Ignace Guillotin announced to the National Assembly of France 116 years earlier. “With my machine, I’ll have your head off in the blink of an eye, and you will suffer not at all” [2]. The Assembly laughed, and while the doctor had not designed the guillotine, the device would forever bear his name [3]. Before this, beheading, typically a death reserved for nobles while hanging was reserved for commoners [2], was a brutal business. Even the most skilled of executioners could botch a beheading, as seen by the unfortunate death of Mary Queen of Scots, who required two blows to be beheaded [4]. One executioner reminded the National Assembly of the Duc de Lally, whose beheading via sword was even more gruesome - he required so many blows that he eventually had to be flipped onto his back to fully sever the head from the body. If many executions were necessary, the use of a sword or axe became even worse, as it was “…absolutely necessary that [the blade] be sharpened again” after each death [2]. The guillotine offered a useful way to circumvent this - with the “mechanization of punishment”, the regime could deliver a humane, egalitarian punishment that did not cause unnecessary pain or torment, did not distinguish between noble and commoner, and could execute up to 20 people per hour [2]. By 1792, the National Assembly voted the guillotine, “the most gentle of lethal methods,” into law [3].

The argument for retained consciousness

It did not take long for the assumption that the guillotine provided painless, instantaneous death to come under challenge. On July 17, 1793, Charlotte Corday was executed via guillotine in front of a curious crowd. Much to the astonishment of the spectators, when the executioner raised up her head and allowed observers to slap it, the cheeks reddened as though she was blushing in indignation. By 1795, physicians such as Samuel Thomas von Sömmerring began to argue that perception may persist in the brain after decapitation - far from being the merciful execution that rendered commoner and noble equal in death, the guillotine was an agonizing torture [3]. Dr. Sömmerring, writing of severed heads grinding their teeth and of facial muscles twitching, speculated that “if air still circulated through the organs of the voice… the heads would speak” [2]. Acting on Sömmerring’s theories, Dr. Heinrich von Leveling conducted several experiments on guillotined victims, mechanically irritating the exposed spinal cord to elucidate grimaces from the beheaded face [3]. Although Sömmerring admitted to regretting his part in these experiments later, he eventually estimated that the “life force” present in the heads lasted for up to 15 minutes, an estimate he obtained from the “retention of heat” in the head [3]. In the 1795 publication *Opinion on the punishment meted out by the guillotine and the pain that continues after severance*, Dr. Jean-Joseph Sue agreed that, from the example of chickens or butterflies that continue to move after decapitation, it is logical to assume that humans retain a torturous awareness after being guillotined, even if only for a moment, such a torture would be “incalculably long” for the victim. He suggested drowning as a possible alternative [3].

In fact, physicians had investigated the possibility of retained consciousness in severed heads even before the revolutionary government officially adopted the guillotine. In 1791, German physicians used electrical probes to evoke “grotesque grimaces” from the severed heads of decapitated criminals, raising the alarming possibility that consciousness possibly persisted after execution [5]. Even in June 1956, Doctors Fournier and Piedelièvre reported to the French Academy of Medicine that death via guillotine “is not immediate… every vital element survives decapitation. The doctor is left with this impression of a horrible experience, of a murderous vivisection, followed by a premature burial” [1]. In a similar vein, Camus tells of Father Devoyod, a chaplain at the infamous Santé Prison who often dealt with the condemned during the mid-20th century. Devoyod recounted one case where, following one execution, he “could see the condemned man’s eyes fixed on me with a look of supplication, as if to ask forgiveness. Instinctively we made the sign of the cross to bless the head, and then the lids blinked, the expression of the eyes softened, and finally the look, that had remained full of expression, became vague” [1].

As more stories such as this circulated, those who supported the guillotine as a humane method of execution grew more forceful in their arguments. In one particularly gruesome experiment, seven condemned criminals were guillotined on November 21, 1803. Immediately after their execution, a number of medical students seized the decapitated heads, shouting at them to provoke a response. No reaction was observed [5]. Members of the National Assembly continued to vehemently argue that death from the guillotine was instant - and even if it wasn’t, the sheer shock of the blow was enough to destroy any semblance of rational thought. Any movements noted were nothing more than the meaningless convulsion of dying muscles [3]. Still other physicians noted that severing the spinal column resulted in instant death in rabbits, chickens, and birds - why not the same in human beings [2]?

But as the years passed, even the arguments for the humaneness of the guillotine became steadily more doubtful. Not even the mechanical marvel, a “material celebration of scientistic [sic] Enlightenment codes… a rational instrument technology par excellence…” [2], was immune to botched executions. On one infamous occasion, the descending blade jammed before it could fully cut through the condemned victim’s neck. The executioner attempted five times to raise the blade and drop it again, until, to the shock of the onlookers, the condemned man managed to wrestle himself free of the device. In the end, he only died when one of the executioner’s assistants “hacked off the head by hand” [6]. This shocking display, which was widely popularized and commented on by figures such as Victor Hugo [6], could not have been more different than the efficient, quick death that the French Ministry of Justice had promised in 1792 [2]. The graphic nature of even successful executions gradually turned public opinion against the device - in many cases, a screen was required to prevent any onlookers from being sprayed with blood [6]. Executions, once proudly displayed to the world during the days of the Revolution at the l’Hotel de Ville in Paris, were gradually moved to the inner courtyards of prisons [1], as the public lost its grim fascination with the guillotine [6].

As the 19th century transitioned into the 20th century, opposition to the guillotine, and the use of the death penalty as a whole, gradually grew into a more and more powerful force within French society [1,6]. The last official death by guillotine was of Hamida Djandoubi, a convicted murderer put to death on September 10, 1977 [6]. His execution was also the last in Western Europe [6]. The few attempts since then to bring back the guillotine as a method of execution, including one proposal by the 1996 Georgia state legislature, which noted the potential viability of organ harvesting from the condemned, have universally failed [6]. However, the scientific debate over the preservation of consciousness in severed heads had already transitioned from humans to animal models by the time of the final guillotining [7].

Just two years before Djandoubi’s death, a 1975 study examining electroencephalogram (EEG) findings in six decapitated rats found that approximately 13.6 seconds of EEG activation followed beheading - a finding that the researchers concluded was consistent with discomfort and pain [7]. This finding was so alarming that the American Veterinary Medical Association (AVMA) soon recommended that decapitation be avoided in laboratory animals, and if necessary, that the severed heads be immediately immersed in liquid nitrogen [8]. A 2013 study found similar results in 10 rats that were decapitated under minimal anesthesia. For an interval of nearly 10 seconds following decapitation, researchers found significant increases in EEG frequency bands that decayed into an isoelectric finding within 15 seconds. The researchers associated the initial jump in the EEG with pain response in the cerebral cortex, followed by a loss of consciousness (LOC), and ultimately concluded that these responses indicate continued nociception post-decapitation [9].

The implication that severed heads may, however briefly, retain the capacity for life has been supported by a number of unusual experiments over the past century in the field of head transplantation. In 1908, Dr. Charles Guthrie performed the world’s first canine head transplant, in which he attached one dog’s head onto the throat of another dog, reconnecting arteries so that the host provided blood flow to the newly-attached head. Of note, this procedure took approximately 20 minutes, and while the transplanted head displayed some simple reflexes, it quickly deteriorated [10]. Dr. Vladimir Demikhov, one of the founders of modern thoracic surgery, repeated a similar experiment in 1954. The heads that he transplanted displayed complex behavior and survived for far longer, up to 29 days, likely because of the significantly shorter time they were without blood flow [10]. Dr. White took the field a step further in 1970 when he performed the first “cephalic exchange transplantation” in primates. Although this transplant involved cervical spine transection of the animals and thus continuous respiratory support, the two heads displayed a normal awake EEG pattern after the surgery [10]. In 2015, Dr. Ping Ren performed a similar experiment with mice, and in one notable example, was able to keep the animals alive for six months [10]. While the science-fiction trope of a "brain in a jar” is impossible for the time being, these experiments clearly demonstrate that the long-term survival of a transplanted head is quite possible. This, in addition to the 1975 [7] and 2013 [9] studies discussed above, suggests that there is no functional difference between the brain of an executed human and the brain of an intact human, for at least several seconds post-decapitation.

The argument against retained consciousness

However, the 1975 report that initially concluded that laboratory rats retained pain perception after decapitation [7] is not without contention. The initial study only had six animals, with a range of 5.6 - 29.5 seconds for the EEG that the researchers interpreted as active [7], a relatively low-powered model. The same phenomenon of EEG activation that the researchers witnessed can also be seen in severed heads under deep anesthesia or in normal brains in deep anesthesia or rapid eye movement (REM) sleep [8]. There is a great deal of debate in the field over this finding, with some papers arguing that this cerebral reaction ultimately does not resemble the typical reaction that rats display in response to painful stimuli [11]. However, a more recent 2013 study concluded that the EEG change seen in rat brains after decapitation - a sharp jump in the median frequency of the EEG - is similar to the EEG changes seen in calves killed by ventral neck incision, suggestive that this is associated with pain perception in a conscious animal [9]. Unfortunately, much like its 1975 predecessor [7], this study was also able to use only 10 animals [9], potentially limiting its use to researchers.

The EEG is not the only potential measurement of brain activity. Using visual evoked potentials (VEP, a focal cortical response to a specific stimulus such as a flash of light), researchers found that decapitation produced a significant reduction in VEP within 10-15 seconds and a significant decrease in EEG amplitude within 15-20 seconds. Further complicating the issue is that the degree of alteration in VEP and EEG amplitude required for loss of consciousness remains unknown. The researchers ultimately concluded that while other methods of euthanasia, such as cervical dislocation and inhalation of carbon dioxide, should be considered “similar to or better than decapitation in their ability to disrupt cortical function,” decapitation remains a humane method of euthanasia in rodent models [12].

The significant amount of exsanguination associated with the use of the guillotine is well-documented [6] and should also be considered when examining animal models. A 1991 study that examined the decapitated rat brain concluded that within 2.7 seconds, the oxygen tension within the organ would decline enough to force the brain into unconsciousness. Thus, the maximum time that pain and distress may be perceived is 2.7 seconds [13]. Since then, this approximate measurement - sometimes increased to three to six seconds - remains the rule of thumb for estimating the time to LOC in decapitated rodents [14,15]. Most tellingly, in the 2000 Report of the AVMA Panel on Euthanasia, the Panel dismissed its earlier concern over decapitation and ruled that it was a humane method of euthanasia for rodent models [12]. Interestingly, Wedekind made a similar argument in 1795, stating that the sheer amount of blood loss in decapitation would produce nearly instantaneous LOC [2].

For obvious reasons, in terms of decapitation, the rodent model does not fully translate to human beings. However, rodent models have long been considered among the most suitable choices for neurotrauma research [16-18]. The possibility of decapitation experiments involving more phylogenetically advanced animal species is considerably more difficult due to financial constraints and a number of other reasons. Humans, when beheaded in the modern day, typically are decapitated mid-neck between the C2 and C5 vertebrae, due to both the weakness and exposed nature of this area [19]. Historical executions via guillotine often ensured ease of decapitation by removing any clothes or fabric in this area that might have hindered the blade [6]. The thick, bony spinal columns that many vertebrates such as pigs possess make them far more difficult candidates for decapitation [20]. Non-mammalian vertebrates are poor candidates as well. In one 2008 study, researchers found that the eel *Anguilla anguilla*, when decapitated, showed signs of life in its severed head for up to eight hours [21], far longer than even the most remarkable anecdotes attributed to the guillotine. Of note, the 2020 AVMA guidelines conclude that for fish and aquatic invertebrates, “decapitation alone is not considered a humane approach to euthanasia, especially for species that may be particularly tolerant of low O2 concentrations” and recommend that any decapitation be followed by immediate pithing of the brain. These same guidelines apply to the CNS of reptiles and amphibians, which may be tolerant to “hypoxic and hypotensive conditions” [22]. Dr. Jean-Joseph Sue raised a similar argument in 1795 when he noted the possibility for certain animals to continue signs of life after decapitation [3]. Ultimately, the current guidelines recommend decapitation as a form of euthanasia for avians/poultry, fish (if followed immediately by pithing or exsanguination), and rodents [22]. It appears unlikely that further experimentation outside of these model animals will proceed in the future.

The beheading of humans is likely at its lowest point in history. Decapitation, when it does occur in the modern day, is more likely to occur via accident or suicide than homicide [19]. Saudi Arabia is the only nation that continues to use beheading as a form of capital punishment [23]; however, unlike the guillotine of the French Revolution, legendary for "the production of rapid and discrete deaths" [2], the condemned are decapitated via a sword called a *sulthan*. As with the historical cases stated above, the condemned often require several blows to be declared dead. One executioner, reflecting on his 35-year-long career and some 600 executions, admitted in a 1989 interview that “in some cases, it has taken two blows for them to die and in very rare cases, three” [23]. At the time of this writing, the Saudi government had executed 130 people in 2022 alone [24]; how many of these were beheaded and how many died via the alternative method, firing squad [23], is impossible to determine.

Ultimately, while the animal models of the twentieth and twenty-first centuries can provide us with some insight into the persistence of consciousness in the severed head, there remain many questions, several of them relating to the nature of consciousness itself. With the limited evidence available to us, it appears safe to agree with the Prussian king's declaration in April 1803 that the facial movements of beheaded men were due to reflexive muscle contractions, not the continued existence of any awareness [5]. The stories from the eighteenth and nineteenth centuries of blushing heads and moving eyes are interesting anecdotes, but in the opinion of this author, they do not constitute scientific evidence. Many of them are plainly exaggerated - Sömmerring’s theories of retained consciousness for up to 15 minutes after decapitation [3] are as outlandish as the legend of the pirate Blackbeard’s headless body swimming around his ship after his death [25]. In a similar fashion, it is difficult to imagine how Charlotte Corday’s decapitated head could *blush *[3] without access to the heart and the circulatory system. Smith states that “it is no accident that morbid popular interest peaked when [Corday] was slapped by the executioner” [2]. Horrified by a bystander slapping a woman, the French government quickly arrested the perpetrator [3]. Primed by the societal mores of the time, it should perhaps come as no surprise when the gathered crowd witnessed evidence of what they perceived to have been “…shame at the infamy of this moral violation” [2]. Ultimately, this widely repeated anecdote may be closer to a morality tale than an actual historical event. The French public’s fascination with the guillotine, for example, it was common for bystanders to dip their handkerchiefs in the blood around the scaffold in a gruesome “Red Mass” [2], likely further exacerbated these rumors.

Many other nineteenth-century tales of decapitated heads can also, on further inspection, be discounted as “fantastic speculation and morbid popular inquiry.” In works such as *The Scaffold and Other Cruel Tales*, authors such as Villiers de I’Isle Adam popularized accounts of experiments conducted on beheaded criminals; while these stories were fictional, they only helped fuel the rumor mill that surrounded the fate of those executed via guillotine [2]. Any scientific efforts to dispel these rumors, such as in Dr. Beauirex’s 1905 experiment, ended up merely “providing food for a popular imagination more attuned to the fantastic and uncanny possibilities” [2]. More modern tales of beheadings via guillotine, such as the 1956 report to the French Academy of Medicine, contradict Dr. Beauriex’s description of the focused and living eyes of the beheaded Languille, noting that “… in the severed head the eyes are motionless with dilated pupils; fortunately they look at nothing" [1]. Similarly, the 1803 experiments on condemned criminals found no evidence of retained life in the executed [5]. In short, for every anecdote of consciousness in severed heads, no matter how nebulous or secondhand, there is a conflicting account of no retaining consciousness.

## Conclusions

The evidence currently available to us is scant, and the studies that imply that there is a retained awareness in decapitated rats for several seconds suffer from a low sample size. While the best evidence currently available to us suggests that LOC is nearly instant in decapitation for both human and rodent models, it is possible that the truth will never be fully known. No government on Earth continues to use the guillotine as a method of execution while beheading via sword is confined to a single nation. The historical accounts that are available to us remain frustratingly scant in evidence and are often closer to urban legends than actual scientific accounts. However, there is a new possibility for further insight into this bizarre historical anomaly. As research into head transplantation continues, and as science continues to probe the edges of human consciousness, a clearer picture may one day emerge.
